# MR Image Reconstruction Using Block Matching and Adaptive Kernel Methods

**DOI:** 10.1371/journal.pone.0153736

**Published:** 2016-04-26

**Authors:** Johannes F. M. Schmidt, Claudio Santelli, Sebastian Kozerke

**Affiliations:** 1 Institute for Biomedical Engineering, University and ETH Zurich, Zurich, Switzerland; 2 Imaging Sciences and Biomedical Engineering, King’s College London, London, United Kingdom; National Taiwan University, TAIWAN

## Abstract

An approach to Magnetic Resonance (MR) image reconstruction from undersampled data is proposed. Undersampling artifacts are removed using an iterative thresholding algorithm applied to nonlinearly transformed image block arrays. Each block array is transformed using kernel principal component analysis where the contribution of each image block to the transform depends in a nonlinear fashion on the distance to other image blocks. Elimination of undersampling artifacts is achieved by conventional principal component analysis in the nonlinear transform domain, projection onto the main components and back-mapping into the image domain. Iterative image reconstruction is performed by interleaving the proposed undersampling artifact removal step and gradient updates enforcing consistency with acquired k-space data. The algorithm is evaluated using retrospectively undersampled MR cardiac cine data and compared to *k-t* SPARSE-SENSE, block matching with spatial Fourier filtering and *k-t ℓ*_1_-SPIRiT reconstruction. Evaluation of image quality and root-mean-squared-error (RMSE) reveal improved image reconstruction for up to 8-fold undersampled data with the proposed approach relative to *k-t* SPARSE-SENSE, block matching with spatial Fourier filtering and *k-t ℓ*_1_-SPIRiT. In conclusion, block matching and kernel methods can be used for effective removal of undersampling artifacts in MR image reconstruction and outperform methods using standard compressed sensing and *ℓ*_1_-regularized parallel imaging methods.

## Introduction

Undersampling of k-space acquisition allows for accelerated MR exams. Partial Fourier methods and parallel imaging exploit redundancy in k-space such as Hermitian symmetry [1] or differences in spatial sensitivity maps of multiple receive coils [2–4] to restore missing k-space profiles. In contrast, reconstruction techniques exploiting redundancy in the image domain depend on the information content in the image data. By exploiting transform properties of correlated image data, undersampling artifacts are removed by filtering in a transform domain. For example, in *k-t* methods [5–7], undersampling artifacts are removed by adaptive filtering of the data in the spatiotemporal frequency domain.

In compressed sensing (CS), incoherent undersampling of k-space is used to introduce noise-like image artifacts which are subsequently suppressed by nonlinear image denoising in a sparse domain while enforcing data consistency with the acquired k-space data [8]. Several sparse linear transformations have been proposed for image reconstruction including Wavelets or image gradients for spatial denoising [8,9], temporal Fourier transform (FT) [10–12], temporal gradients [13], time-frame reordering [14], low-rank or principal component transformations along time [12,15–17] and combinations thereof [18]. In addition, data-dependent transformations can be included and optimized during reconstruction by dictionary learning [19,20] and blind CS [21,22]. Sparse representations of image patches can also be exploited along spatial [23] and temporal [12,24,25] directions.

Nonlinear transforms for MR image reconstruction have also been used for nonlinear GRAPPA [26] where the nonlinearity in the bias between ground truth and noisy GRAPPA coefficients is modeled with a polynomial kernel and transformation into a higher dimensional feature space. For dynamic imaging, CS reconstruction in a feature space with linear and quadratic terms motivated by a second degree polynomial kernel allowed for higher undersampling factors for ASL perfusion data sets [27]. Further work included kernels with radial basis functions [28] and self-learned nonlinear dictionaries [29] for enhanced sparsity in time domain.

In the present work, suppression of incoherent undersampling artifacts by linear projection of nonlinearly transformed image block arrays is proposed. In each iteration, the current image estimate is subdivided into overlapping blocks. Each block is grouped with matching blocks from the image based on a preceding clustering analysis. The block array is transformed according to nonlinear Gaussian weights assigned to each block where the mapping is implicitly calculated based on kernel PCA with a Gaussian kernel. Denoising in the nonlinear domain is achieved by projection onto the most significant principal components followed by a back-mapping into the image domain. MR image reconstruction is performed by iteratively interleaved gradient updates for consistency with the acquired k-space data and denoising in the kernel feature space. The efficacy of the reconstruction is evaluated on two-dimensional cine data of the heart.

### Theory

#### Image reconstruction by denoising of matching image blocks

**Fig 1:**
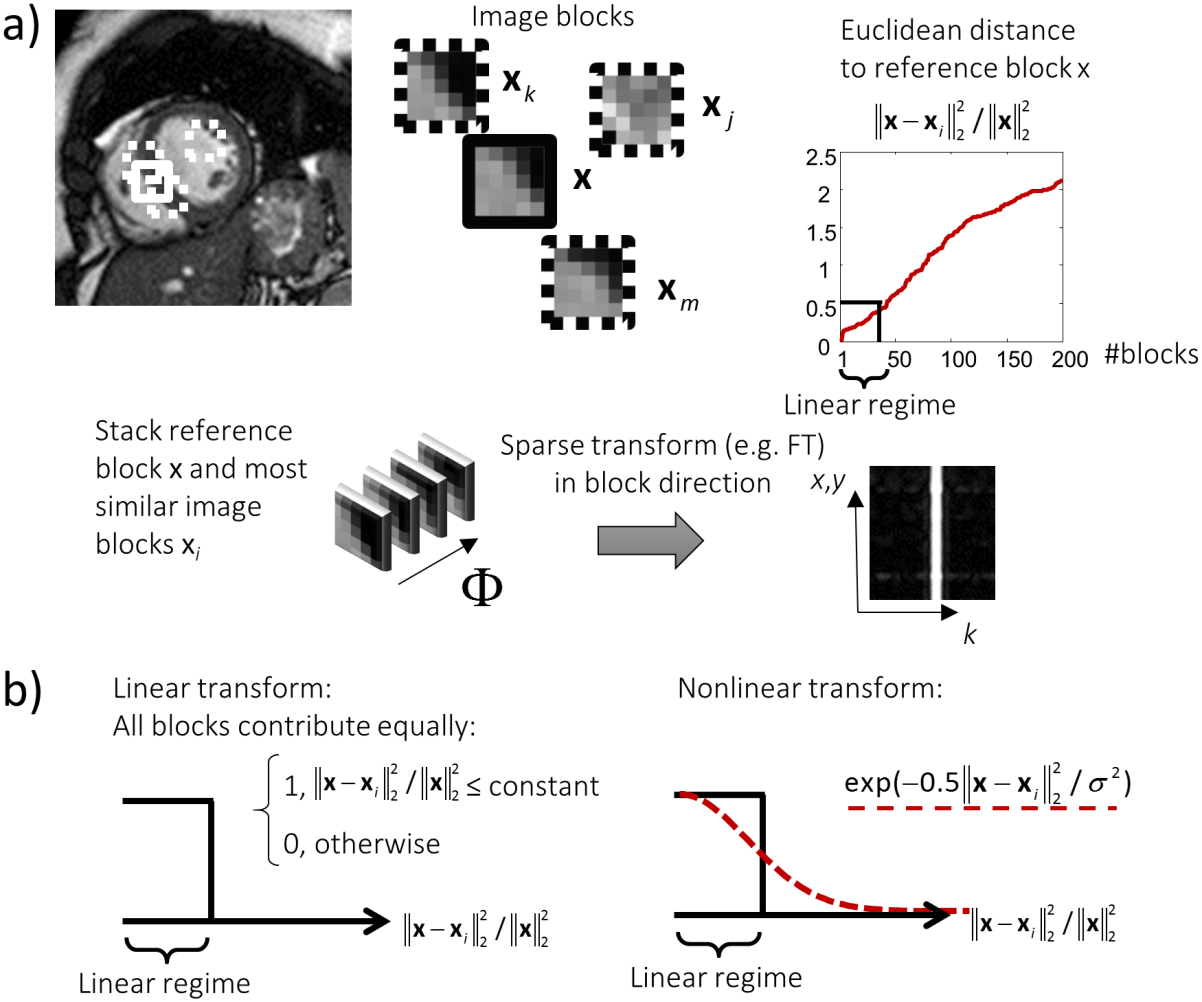
**a)** Grouping of similar sub-blocks obtained from an image allows for enhanced sparsity. A reference image block **x** is denoised by stacking it along with similar image blocks **x**_*i*_ into a multi-dimensional array and performing collaborative filtering in a transform domain. An upper cut-off criterion for the maximum distance between reference block **x** and adjacent blocks determines how many image blocks are used for the transform; all image blocks contribute equally (linear regime). **b)** Nonlinear methods can be used for transforms where image blocks contribute depending on a nonlinear function, e.g. a Gaussian function.

CS image reconstruction relies on iterative image denoising while ensuring consistency with the acquired k-space data. Early implementations employed algorithms with explicit or implicit assumptions on the underlying image such as being piece-wise constant for total variation based denoising or being smooth with a small set of discontinuities in Wavelet based image reconstruction algorithms [8]. Advanced techniques employ overcomplete dictionaries [19,20] or data-dependent transforms based on image patches to preserve image details and reduce smoothing artifacts by exploiting redundancy in substructures of image blocks [30]. To denoise a reference image block **x**, image blocks are sorted according to a similarity measure, e.g. based on the Euclidean distance (Fig 1a). By choosing an upper cut-off criterion, all similar image blocks are stacked and transformed in stack direction using a sparse transform, for example using the FT or a singular value decomposition. Each image block contributes equally to the transform and the upper cut-off only allows for a limited number of blocks to be used. If there are only a few image blocks with high similarity, the transform domain sparsity is deteriorated. Adding more blocks with lower similarity leads to denoising artifacts and smoothing.

These limitations can be mitigated using nonlinear transforms where data-dependent transforms can be composed of all available image blocks. Employing kernel principal component analysis (PCA) with a Gaussian kernel, for example, the contribution of each image block to the transform depends on the mutual distances in a nonlinear way. Image blocks with high similarity relative to **x** contribute more to the transform and blocks with lower similarity contribute less, but there is no need for an upper cut-off (Fig 1b). An introduction to kernel PCA is given in the next paragraph.

#### Kernel PCA

**Fig 2:**
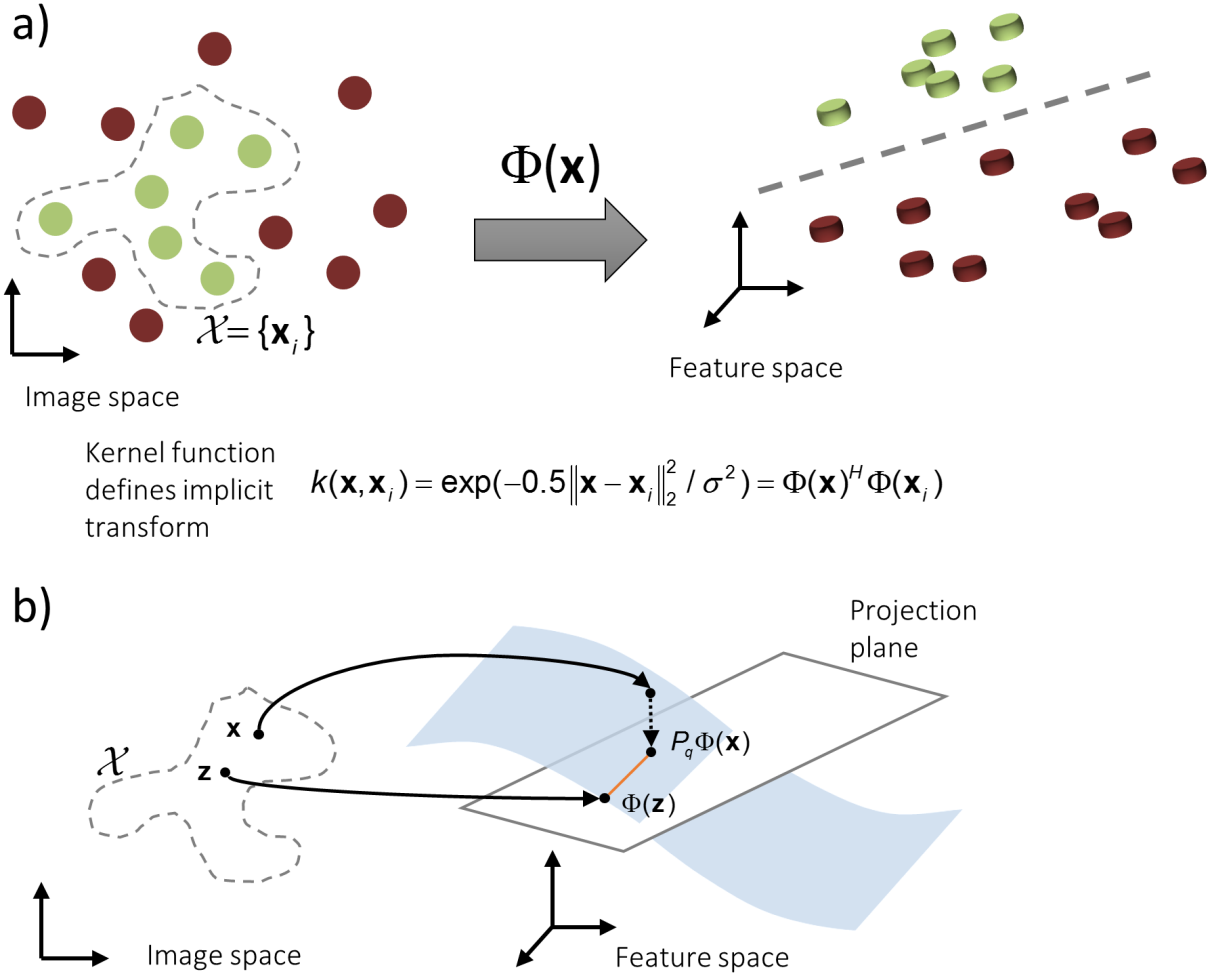
**a)** Kernel PCA deduces an implicit transformation Φ from input space X (green circles) into a high-dimensional feature space where linear algorithms can be employed to separate image data from artifacts (red circles). **b)** Denoising is performed by projecting the test vector **x** onto the first *q* principal components by *P*_*q*_. Backmapping of the projected data is done by finding a so-called pre-image **z** in image space which minimizes the Euclidean distance between Φ(**z**) and *P*_*q*_Φ(**x**).

Kernel PCA [31] is a nonlinear extension to PCA where a linear analysis is performed in a high-dimensional nonlinear feature space. It comprises of a three step process including (1) nonlinear data mapping into feature space where data is linearly separable (Fig 2a), (2) a conventional linear PCA to project data onto the first *n* eigenvectors, and (3) back-mapping of data points from feature space to input space by numerical inversion of the implicit transformation (Fig 2b).

In this work, multi-dimensional image blocks are stacked to vectors composing the kernel PCA input space *χ*. The nonlinear mapping Φ:*χ*→*ℱ* from input space *χ* to the high-dimensional feature space *ℱ* is not calculated explicitly. Instead, kernel PCA reformulates standard PCA in feature space to operate on scalar products of function values Φ(**x**)^*H*^Φ(**y**). The dot products are evaluated directly in input space by means of Mercer’s theorem, which states that any positive semi-definite, symmetric, and continuous kernel function *k*:*χ*×*χ*→ℝ can be written as an inner product *k*(**x**_*i*_,**x**_*j*_) = Φ(**x**_*i*_)^*H*^Φ(**y**_*j*_)

Kernel PCA can then be performed by solving the kernel eigenvalue problem $M\lambda \alpha =\tilde{K}\alpha$, where *M* is the number of data vectors (image blocks **x**_*i*_) in the input space and λ the eigenvalue to the eigenvector **α**. $\tilde{K}$ is given by centering [32] the kernel matrix *k*_*ij*_ = *k*(**x**_*i*_,**x**_*j*_) defined by all entries in the input space *χ* = {**x**_*i*_}. The projection of a centered feature space vector $\tilde{\Phi }(x)$ onto the *n*’th principal component is given by $\beta_{n}=\sum_{i}^{M}\alpha_{n,i}{}^{}\tilde{k}(x,x_{i})$ [33] where **α**_*n*,*i*_ is the *n*’th component of the eigenvector **α**_*i*_. The projection onto a subspace of principal components spanned by the first *q* eigenvectors can be written as $P_{q}\Phi (x)=\sum_{n}^{q}\beta_{n}\sum_{i=1}^{M}\alpha_{n,i}^{}\tilde{\Phi }(x_{i})+\bar{\Phi }=\sum_{i=1}^{M}\gamma_{i}\tilde{\Phi }(x_{i})+\bar{\Phi }$ where $\bar{\Phi }$ is the mean of the mapped data and $\gamma_{i}=\sum_{n}^{q}\beta_{n}\alpha_{n,i}^{}$.

For large numbers of data vectors in the input space and high-dimensional kernel mappings, the nonlinear mapping Φ has typically no analytical inverse function [34]. Approximate solutions can be found by mapping an estimate **z** in the input space to the feature space and update it by optimizing a cost function for the best fit to the projected test value *P*_*q*_Φ(**x**). In this study, an iterative pre-image algorithm [33] is used which minimizes the Euclidean distance in feature space ||Φ(**z**)−*P*_*q*_Φ(**x**)||_2_ with a fixed-point iteration scheme. For any kernel of the form *k*(**x**,**y**) = k(||**x**−**y**||_2_) as given for Gaussian kernels, the iteration steps can be written as

$$z_{t+1}=\frac{\sum_{i=1}^{M}\gamma_{i}k(z_{t},x_{i})x_{i}}{\sum_{i=1}^{M}\gamma_{i}k(z_{t},x_{i})\;}$$ (1)

#### Linear versus nonlinear regime with Gaussian kernels

Throughout this paper, a Gaussian kernel $k(x_{i},x_{j})=\text{exp}(-0.5{\left\|x_{i}-x_{j}\right\|}_{2}^{2}/\sigma^{2})$ is used. To account for the dependency of the Euclidean distance ||**x**_*i*_−**y**_*j*_||_2_ on the square root of the number of pixels per vector, the kernel function can be written as $k(x_{i},x_{j})=\text{exp}(-0.5{\left\|x_{i}-x_{j}\right\|}_{2}^{2}/(N\;\cdot \sigma_{p}{}^{2}))$ where $\sigma_{p}=\sigma /\sqrt{N}$ and σ_*p*_ is the average distance per pixel [33]. The kernel width σ controls the degree of nonlinearity of the mapping and indicates how well the test data match the input space data *χ* = {**x**_*i*_} [35]. In the linear limit of a very large σ, the kernel matrix ***k***_*ij*_ = *k*(**x**_*i*_,**x**_*j*_) comprises only of ones and all data contribute equally to the transform just as for linear transforms. In the nonlinear limit of overfitting where *σ* ≪||**x**_*i*_−**x**_*j*_||_2_,∀**x**_*i*_,**x**_*j*_∈*χ*, the kernel matrix approaches the identity matrix with the canonical basis vectors as eigenvectors. In this case, the test vector **x** is mapped to the vector in the input space {**x**_*i*_} with minimal Euclidean distance. Accordingly, the kernel width should match the scale of the structure which should be denoised [36].

#### Kernel PCA input space and back-mapping to an image

**Fig 3:**
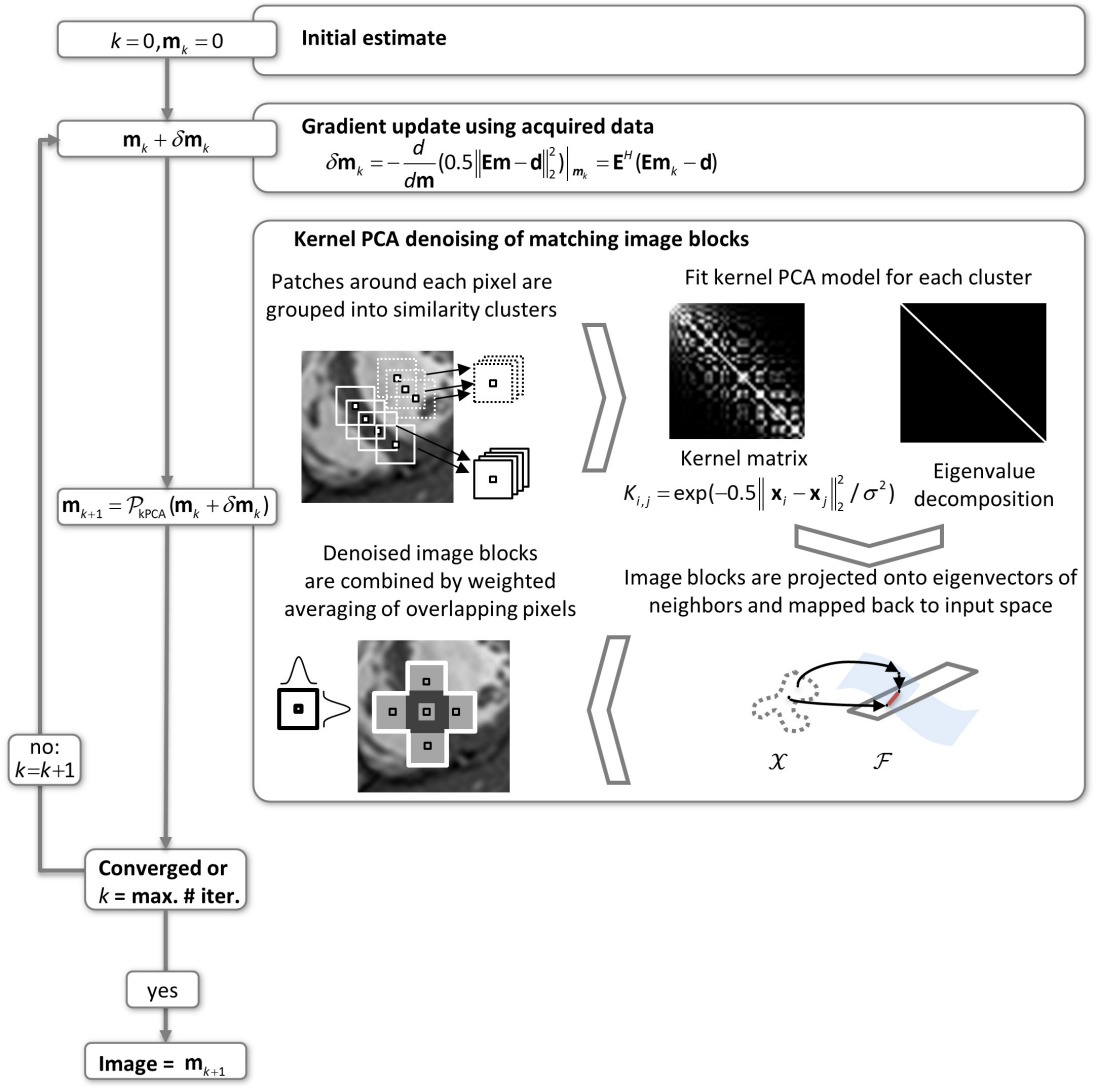
Workflow for image reconstruction with nonlinear kernel PCA. Each iteration consists of two steps: (1) a gradient update ensures consistency with the acquired k-space data, and (2) kernel PCA denoising of image blocks.

In the present work, the temporal mean of the dynamic data set is removed prior to kernel PCA computations to increase the similarity between image blocks. The current image estimate is subdivided into overlapping image blocks which are stacked to vectors **x**_i_. To reduce computational complexity and allow for tailored parameters, the blocks are grouped into *N* clusters using a similarity cluster analysis [37]. A kernel PCA input space is generated for each input cluster using a maximum number of *M* randomly selected input space vectors from the cluster. The dimension of the input space is given by the number of voxels per block (Fig 3). An *M* x *M* kernel matrix is then populated with the selected image blocks using a Gaussian kernel function and a PCA of the kernel matrix is performed. Each image block from the subdivision is finally projected onto the first few principal components in the features space spanned by the *M* image blocks from the input space and subsequently mapped back to the input space using the fixed-point iteration scheme of Eq (1). The filtered image blocks are multiplied with a normalized 3D Gaussian shape function and then added together according to the voxel locations to form the next estimate of the dynamic data set.

#### Projected MR reconstruction for nonlinear transform domains

MR reconstruction inverts a linear encoding equation

d=Em (2)

where **d** are the acquired k-space data, **E** the encoding matrix including Fourier sampling and weighting with receive coil sensitivities, and **m** the image to be reconstructed. Image reconstruction is motivated by iterative thresholding algorithms [38,39] and consists of iteratively interleaved steps of gradient updates from the acquired k-space data and nonlinear denoising. The basic update rule is given by

$$m_{k+1}=P_{\text{kPCA}}(m_{k}-\;E^{H}(Em_{k}-d))$$ (3)

where $P_{kPCA}$ represents the kernel PCA denoising for each image block and **m**_*k*_ the current image estimate.

**Algorithm 1**: Pseudo-code implementation of iterative reconstruction with block-based kernel PCA denoising for dynamic data sets.

 Initialization:

 Choose parameters: image block size **w** = (*w*_*x*_,*w*_*y*_,*w*_*t*_), number of similarity clusters *N*, size of kernel matrix (*M* x *M*).

 1) Get initial image **m**_*0*_ by performing 10 iterations with *k-t* SPARSE-SENSE [40].

 2) Remove temporal mean.

 3) Divide image into overlapping blocks with width **w**.

 4) Perform a k-means cluster analysis to group image blocks into *N* clusters and remember the cluster centers and the cluster of each image block.

 For each *k* = 0,1,2, … repeat until converged or maximum number of iterations reached.

  1) Perform **gradient update** enforcing data consistency ${\tilde{m}}_{k+1}=m_{k}-\;E^{H}(Em_{k}-d)$.

  2) **Image block denoising**: $m_{k+1}=P_{\text{kPCA}}({\tilde{m}}_{k+1})$

   (a) Remove temporal mean.

   (b) Divide image into overlapping blocks with width **w**.

   (c) Refine k-means cluster analysis with an additional iteration.

   (d) For each cluster, randomly select a maximum number of *M* image blocks to build a kernel matrix. Perform **kernel PCA denoising** for each image block **x**_*i*_ in the cluster.

   (e) Compose denoised image by multiplying each block with a normalized 3D Gaussian shape function and summing up image block values corresponding to each image voxel.

   (f) Add temporal mean from step (a).

  3) *k* = *k* + 1.

### Methods

#### Data acquisition

Six fully sampled two-dimensional cine data set in short axis view of the heart were acquired on a 3.0 T scanner (Ingenia, Philips Healthcare, The Netherlands) with a 28-channel coil array. The scan parameters of the balanced SSFP sequence included a field-of-view of 270x270 mm^2^, 8 mm slice thickness, TR/TE of 3.8/1.84 ms, voxel size of 1.4x1.4 mm^2^, 45° flip angle, 192x190 acquisition matrix, and 23–28 heart phases. Noise samples were acquired prior to the scans to calculate the noise covariance matrix. All data were acquired in healthy subjects after written consent was obtained according to institutional and ethics guidelines. The study protocol was approved by the ethics committee of the canton of Zurich.

#### Data preparation

All k-space data sets were pre-whitened with the noise covariance matrix [41] and normalized to a mean signal strength of 1 in the region-of-interest (ROI) around the heart. k-space data were compressed to 12 virtual coils [42] and normalized coil sensitivity maps were obtained with ESPIRiT [43]. Retrospective undersampling was performed in phase encoding direction using Cartesian pseudo-random undersampling [8]. Undersampling factors were 5, 6.5 and 8.

#### Data reconstruction

The cine 2D data sets were reconstructed with *k-t* SPARSE-SENSE [40], *k-t ℓ*_1_-SPIRiT [44], block matching with Fourier filtering similar to LOST [23] and the proposed algorithm.

The *k-t* SPARSE-SENSE algorithm minimizes

$$||d-Em|{|}_{2}^{2}+\lambda ||{ℱ}_{t}\;m|{|}_{1}$$ (4)

with **d** and **E** as defined in Eq (2), and *ℱ*_*t*_ being the temporal FT. The regularization parameter λ was chosen to minimize the RMSE in the ROI of an exemplary data set.

In the *k-t* *ℓ*_1_-SPIRiT reconstruction of the cine 2D data, the term

$${\left\|d-E^{S}m^{S}\right\|}_{2}^{2}+\lambda_{1}{\left\|(G-I)m^{S}\right\|}_{2}^{2}+\lambda_{2}{\left\|{ℱ}_{t}\;m^{S}\right\|}_{1}$$ (5)

was minimized with the Fourier sampling matrix **E**^S^, the multi-coil image **m**^S^, the coil-wise temporal FT *ℱ*_*t*_, and **G** being the image domain representation of the 7x7 k_x_-k_y_ SPIRiT [42] interpolation kernel derived from a 30x20 temporally averaged center of k-space. Eqs (4) and (5) were minimized using an iterative soft-thresholding [39] and a projection onto convex sets algorithm [45], respectively, both leaving the acquired data unchanged. The final image **m** was composed by Roemer combination of the multi-coil images **m**^S^. Thresholds for the soft-thresholding operations were chosen based on minimum RMSE in the region-of-interest of an exemplary data set.

Reconstruction with block matching and Fourier filtering involved the same parameters and clustering algorithm as for the proposed algorithm. The kernel PCA filtering was replaced with soft thresholding in a Fourier domain similar to LOST. The number of blocks per cluster was restricted to a maximum of 24 to reduce smoothing artifacts.

For the proposed algorithm, each iteration consisted of a data consistency and a kernel PCA denoising step comprising of 600 clusters (*N*) of image blocks, a maximum number of 120 blocks (*M*) to generate the kernel matrix per cluster and a block size of 5 pixels in each spatial dimension while using all time frames along the time dimension. The kernel width of the proposed kernel PCA filtering approach was fixed to the median of the mutual distances between the 25 closest image blocks within each cluster to achieve linear filtering between the most similar blocks and increasingly less contribution for blocks with lower similarity. The number of retained principal components was determined per image block cluster based on a two-component model for the cumulated energy in the principal components [36], with a maximum number of 20 retained principal components. Computation time for the clustering refinement was between 1s and 5s, for kernel PCA artifact removal 5s–15s per iteration on current computer hardware with 8 cores.

## Results

**Fig 4:**
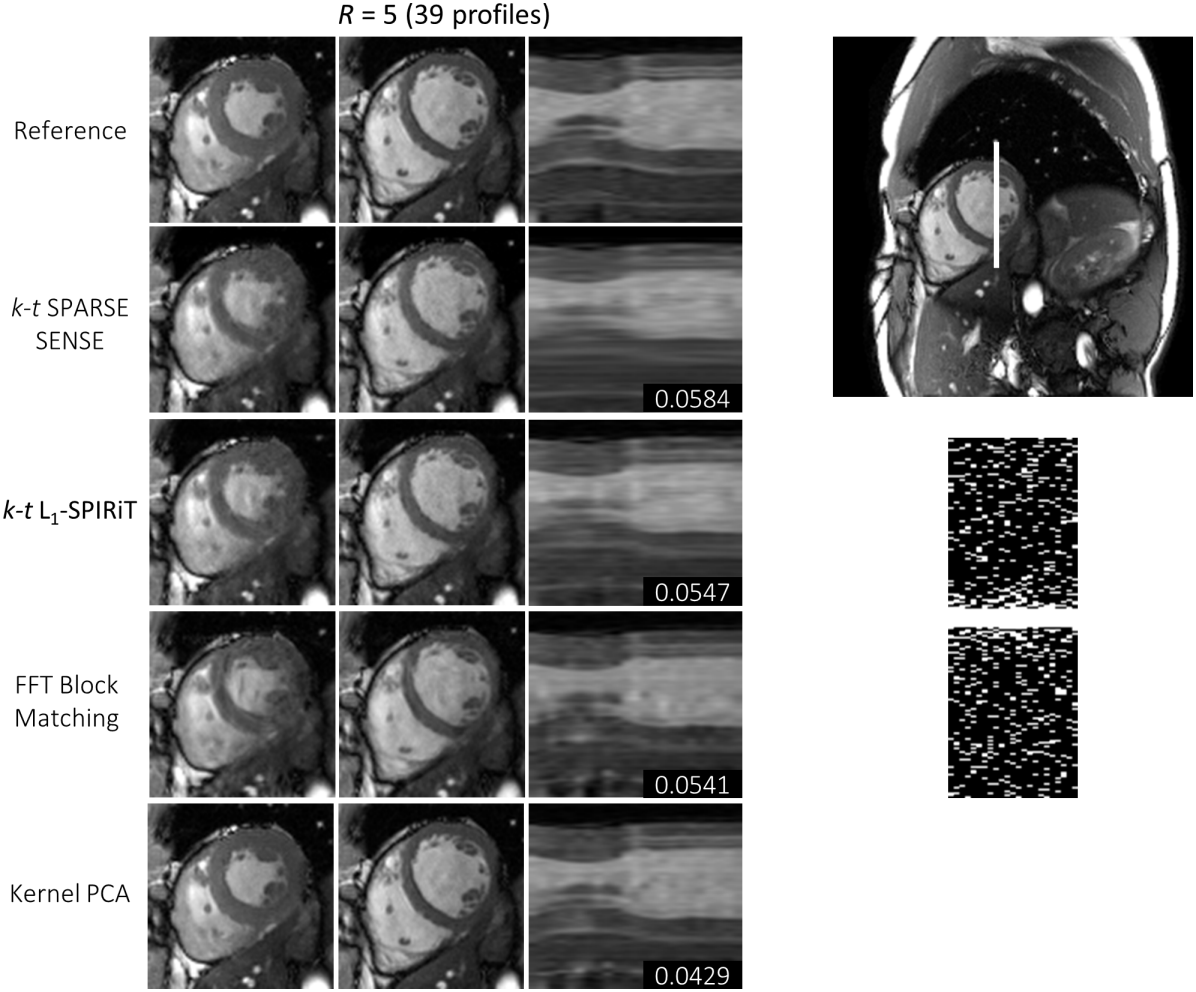
Systolic and diastolic reference data and reconstruction results for 2D dynamic data with a reduction factor of 5. Temporal profile plots are taken along the indicated line. RMSE values for the shown 3D subvolume covering the heart over time are quoted. The figures on the right show the full field of view of the data set as well as the sampling pattern.

**Fig 5:**
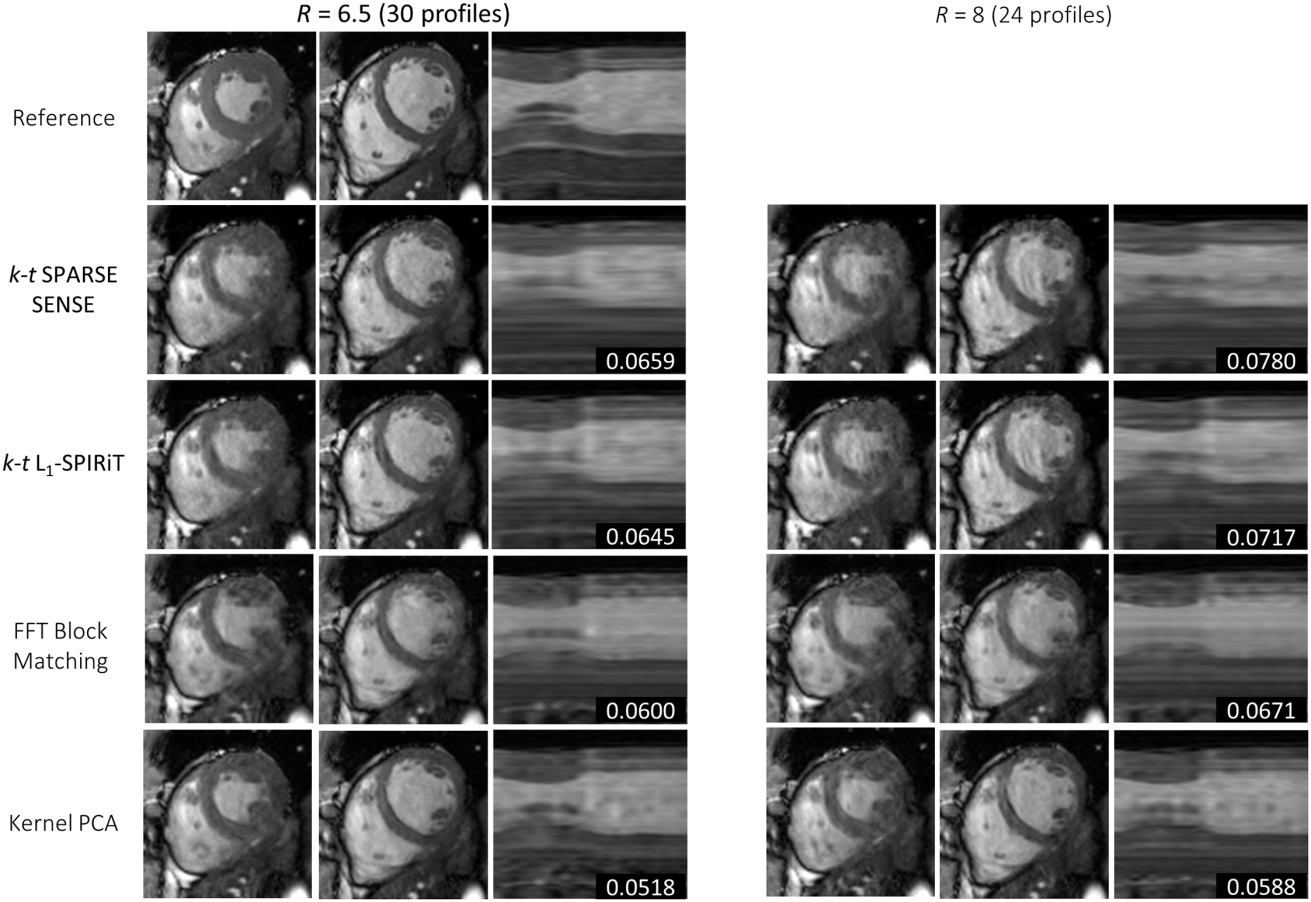
Reference and reconstruction results for 2D dynamic data with reduction factors of 6.5 and 8. RMSEs for the shown 3D subvolume covering the heart over time are indicated.

**Fig 6:**
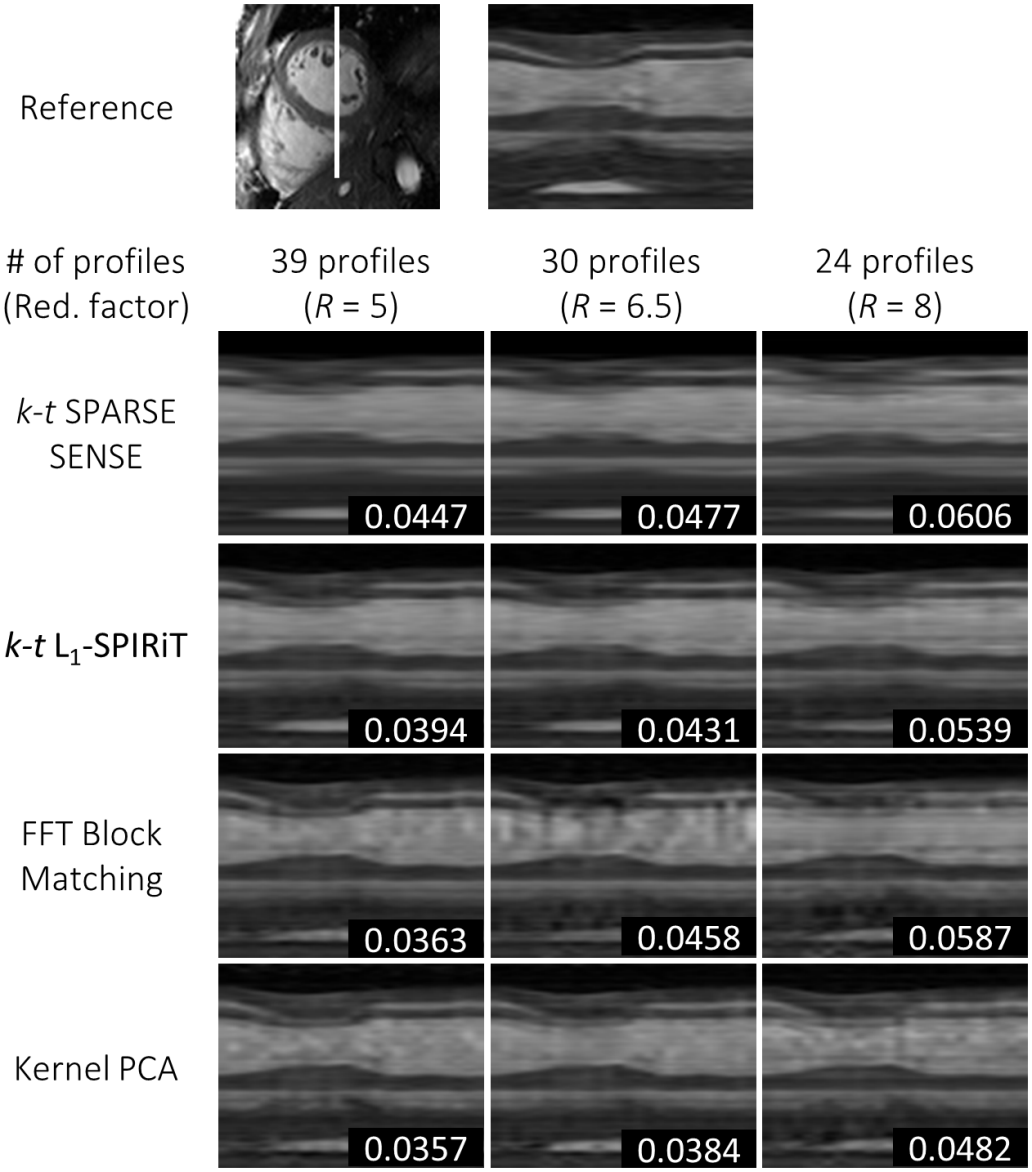
Signal-intensity profiles through the left ventricle for reduction factors 5, 6.5 and 8. RMSEs for a 3D subvolume covering the heart over time are indicated.

**Fig 7:**
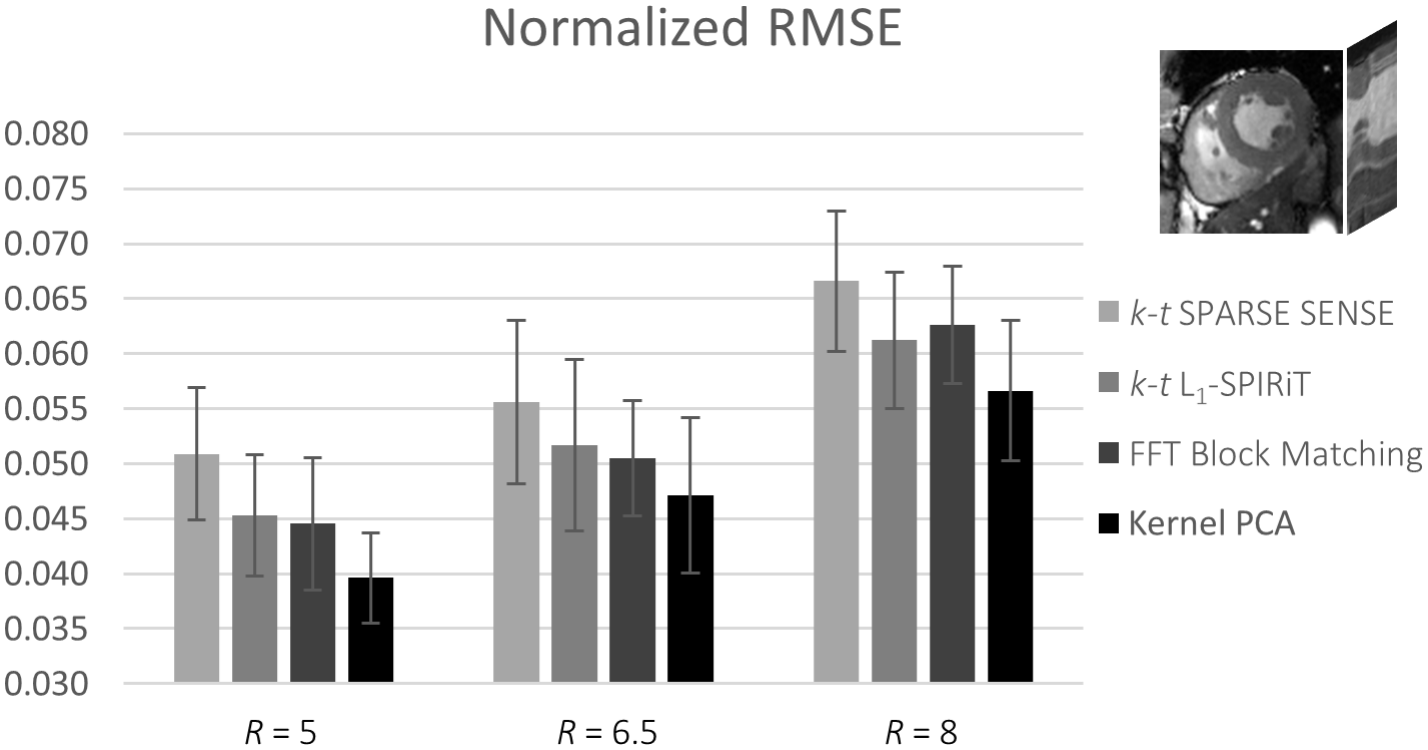
Mean and standard deviation of the RMSE relative to the fully sampled reference for all 2D cine data sets (# of volunteers = 6) in a 3D ROI around the heart as indicated in the upper right corner.

Results for one exemplary cine data set comparing *k-t* SPARSE-SENSE, *k-t ℓ*_1_-SPIRiT with a temporal FT sparsifier, block matching with Fourier filtering, and the proposed kernel PCA reconstruction relative to the fully sampled reference are shown in Fig 4 for 5-fold undersampling. Image quality is compared for systolic and diastolic still frames as well as using temporal profile plots. The proposed algorithm shows less smoothing artifacts especially in the time dimension. RMSE values relative to the reference were determined in the 3D ROI as indicated. Fig 5 compares the different reconstructions for 6.5 and 8-fold undersampling. More image reconstruction results are shown in Fig 6. Mean and standard deviation of the RMSE for all six data sets and the four reconstruction algorithms are visualized in Fig 7. Reconstruction based on kernel PCA is found to exhibit the lowest RMSE.

## Discussion

In this work, an algorithm for image reconstruction from undersampled MR data exploiting block-matching and nonlinear kernel PCA has been proposed and implemented. Images were reconstructed iteratively by interleaved gradient updates using the acquired k-space data and shrinkage of nonlinearly transformed image block arrays. Undersampling artifacts in two-dimensional cardiac cine MR data were reduced and results compared favorably relative to those obtained with other CS-based reconstruction methods.

Compared to linear transforms of image block arrays, the contribution of each image block to the transform with the proposed kernel PCA approach is given by a nonlinear function. The transform is implicitly calculated by kernel PCA and the artifact removal is performed by projection onto the main principal components in the nonlinear transform domain. Gaussian kernels employ the Euclidean distance as dissimilarity measure. Better results may be achieved using dissimilarity measures which are more suitable for MR images, if the kernel function fulfils Mercer’s condition.

The kernel width determines how each image block in the kernel PCA input space contributes to the transform. By choosing the median of the mutual distances of the most similar image blocks as kernel width σ, image blocks with high similarity are filtered linearly, while image blocks which are above the cut-off distance for linear filtering contribute less. The maximum number of retained principal components is calculated per cluster and based on a two-component model.

PCA in feature space is based on a *ℓ*_2_ penalty function which is sensitive to outliers. Especially for high reduction factors, large and correlated undersampling artifacts can already differentiate the first few principal components from the desired ones. Pre-filtering of data and employing statistically robust linear feature selection in feature space [46] could further improve artifact removal and simplify the selection of kernel width and number of principal components.

Iterative thresholding algorithms have computationally economic iteration steps but require more iterations until convergence than gradient descent *ℓ*_1_ minimization [47]. An adaption of approximate message passing [47] can reduce the maximum number of iterations and reduce reconstruction times. Further improvements in reconstruction speed can be achieved by correlating multiple image blocks at once and employing iterative kernel PCA schemes such as the kernel Hebbian algorithm [48], which also scales linearly with the sample size. The use of many computer nodes in parallel, as for example available on graphics cards, could further reduce reconstruction times. The convergence rate of the reconstruction could be increased by modifying the gradient updates with prior knowledge or gradient directions from previous iteration steps [47,49].

## Conclusion

Image reconstruction from undersampled data exploiting nonlinear transform domains and kernel methods is feasible and outperforms conventional *k-t* SPARSE-SENSE, block matching with Fourier filtering and *k-t ℓ*_1_-SPIRiT reconstruction. The method holds considerable potential to allow for higher acceleration factors relative to CS for a range of MR applications including cardiovascular imaging.
